# The Volume-Regulated Anion Channel LRRC8/VRAC Is Dispensable for Cell Proliferation and Migration

**DOI:** 10.3390/ijms20112663

**Published:** 2019-05-30

**Authors:** Tianbao Liu, Tobias Stauber

**Affiliations:** Institute of Chemistry and Biochemistry, Freie Universität Berlin, 14195 Berlin, Germany; tianbao.liu@fu-berlin.de

**Keywords:** LRRC8A, VRAC, cell proliferation, cell migration, glioblastoma, Akt signaling

## Abstract

Cells possess the capability to adjust their volume for various physiological processes, presumably including cell proliferation and migration. The volume-regulated anion channel (VRAC), formed by LRRC8 heteromers, is critically involved in regulatory volume decrease of vertebrate cells. The VRAC has also been proposed to play a role in cell cycle progression and cellular motility. Indeed, recent reports corroborated this notion, with potentially important implications for the VRAC in cancer progression. In the present study, we examined the role of VRAC during cell proliferation and migration in several cell types, including C2C12 myoblasts, human colon cancer HCT116 cells, and U251 and U87 glioblastoma cells. Surprisingly, neither pharmacological inhibition of VRAC with 4-[(2-Butyl-6,7-dichloro-2-cyclopentyl-2,3-dihydro-1-oxo-1H-inden-5-yl)oxy]butanoic acid (DCPIB), carbenoxolone or 5-nitro-2-(3-phenylpropyl-amino)benzoic acid (NPPB), nor siRNA-mediated knockdown or gene knockout of the essential VRAC subunit LRRC8A affected cell growth and motility in any of the investigated cell lines. Additionally, we found no effect of the VRAC inhibition using siRNA treatment or DCPIB on PI3K/Akt signaling in glioblastoma cells. In summary, our work suggests that VRAC is dispensable for cell proliferation or migration.

## 1. Introduction

The volume-regulated anion channel (VRAC), alternatively named the volume-sensitive outwardly rectifying (VSOR) anion channel, is ubiquitously expressed in almost all vertebrate cells [1,2,3]. The VRAC is formed by heteromers of LRRC8 proteins, with LRRC8A being the only essential subunit which has to combine with at least one other LRRC8 member (B–E) [4,5,6]. The channel opens upon osmotic cell swelling and contributes to regulatory volume decrease (RVD) by releasing chloride ions and various organic osmolytes [1,2,3,7,8,9]. The VRAC is additionally involved in further processes beyond RVD, such as cancer drug uptake [10], insulin secretion [11,12], astrocyte-neuron communication [13,14,15], and sperm development [16].

During cell proliferation, the transient activation of Cl^−^ channels leads to a decrease in cell volume after an initial volume increase [17,18,19]. Cell migration is mainly mediated by cytoskeletal rearrangements and directed membrane transport. In addition, osmotic water flux by the differential activity of ion channels and transporters mediating local changes in cell volume was found to contribute to cell movement [20,21]. The uptake of inorganic ions and water (a regulatory volume increase, RVI) at the leading edge by locally active Na^+^-K^+^-2Cl^−^ cotransport, Na^+^/H^+^ exchange or nonselective cation channels, and a volume decrease at the trailing end via release of K^+^ and Cl^−^ through activated K^+^ and Cl^−^ channels followed by water efflux (RVD) will lead to a net translocation of the cell [22]. Recently, cell displacement was found to be solely driven by directed cellular osmotic water transport in an artificial confined environment when actin polymerization was inhibited [23].

Due to its role in regulatory volume decrease, the VRAC has been proposed to be involved in cell volume changes during cell proliferation and migration [17,22,24,25,26]. Consistently, maximal VRAC currents were found to change during cell cycle progression [27]. Indeed, various (rather unspecific) VRAC inhibitors were reported to impair the proliferation rate of a wide range of cell types [28,29,30,31,32,33,34,35,36,37,38]. Glycine-induced cell swelling, on the other hand, was correlated with the migration of microglial cells [39]. The motility of nasopharyngeal carcinoma and glioma cells was reported to be suppressed by VRAC inactivation with pharmacological inhibitors or hypertonic solution. [24,25]. More recently, the VRAC inhibitor 4-[(2-Butyl-6,7-dichloro-2-cyclopentyl-2,3-dihydro-1-oxo-1H-inden-5-yl)oxy]butanoic acid (DCPIB) was shown to reduce the migration of glioblastoma cell lines [28]. The siRNA-mediated knockdown of the obligate VRAC subunit LRRC8A was reported to limit the proliferation of glioblastoma cells [40] and reduce the migration of human colon cancer HCT116 cells [41].

However, several observations cast doubt on a general role for the VRAC in cell proliferation and migration. So far, no proliferation defect has been reported for any of the various published LRRC8A-deficient cells lines. The proliferation of HeLa cells was reported to be unaffected by the siRNA-mediated knockdown of LRRC8A [42]. Recently, the flavonoid Dh-morin was shown to effectively inhibit endogenous VRAC currents in endothelial cells, without impairing the proliferation of human umbilical vein endothelial (HUVEC) cells [43], arguing against a crucial role for the VRAC in the cell cycle progression of this cell type. The anti-proliferative effect of submicromolar concentrations of cardiac glycosides was even linked, albeit not necessarily directly, to an increase of VRAC activity in HT-29 colorectal cancer cells and could be blocked by the VRAC inhibitor DCPIB [44].

In all, there are conflicting data as to the role of VRAC in cell proliferation and migration. To study the potential role of VRAC in cell proliferation and migration, and therefore we systematically examined these processes for a variety of cell lines, including cancer and noncancer cell lines, by using pharmacological blockers, siRNA against LRRC8A, and genomic VRAC knockout. In none of the studied cell lines did we find evidence for a critical involvement of VRAC in cell proliferation or migration.

## 2. Results

### 2.1. LRRC8A Knockout Does Not Impinge on the Proliferation and Migration of C2C12 Cells

**Figure 1:**
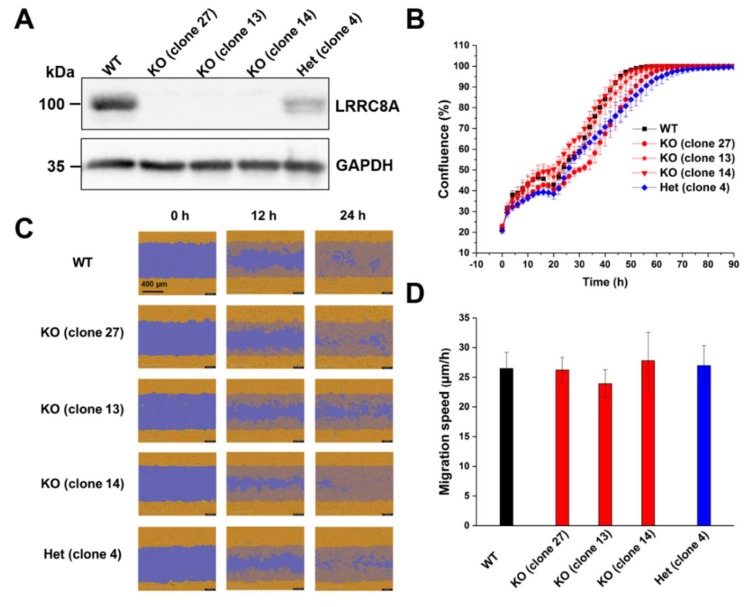
LRRC8A depletion does not affect C2C12 cell proliferation and migration. (**A**) Protein levels of LRRC8A in wild-type (WT), LRRC8A-knockout (KO) and heterozygous (Het) C2C12 cells tested by Western blotting. GAPDH used as loading control. A representative blot of three independent experiments is shown. (**B**) Growth curve of WT, KO, and Het C2C12 cells. Data represent mean ± SD of *n* = 4 experiments. (**C**) Representative images of the wound healing assay with C2C12 cells at indicated time points. Initial wound mark depicted in blue, cells in orange on blue overlay represents migrated cells. Scale bar, 400 μm. (**D**) Quantification of migration speed of C2C12 cells. Cell migration speed was determined 14 h post-wounding. Data represent mean ± SD of *n* = 14 experiments.

To investigate the putative role of the VRAC in the proliferation and migration of C2C12 mouse myoblast cells, we used various clonal genome-edited cell lines deficient for the essential VRAC subunit LRRC8A (clones 27, 13, and 14) and a line (clone 4) with only heterozygous LRRC8A deletion that had experienced the same transfection and selection process as the knockout clones. The loss of LRRC8A in the knockout clones and reduced levels in clone 4 in comparison to wild-type C2C12 cells was confirmed by Western blotting (Figure 1A). We first assessed the effects of LRRC8A knockout on C2C12 proliferation. The proliferation rate of the knockout clones was similar to that of wild-type cells or the heterozygous clone (Figure 1B), demonstrating that VRAC is dispensable for C2C12 cell proliferation. Next, we investigated the role of LRRC8A in cell migration using a wound healing assay (Figure 1C,D). We observed no significant differences in migration speed among LRRC8A knockout (clone 27, 26.22 ± 2.15 μm/h; clone 13, 23.91 ± 2.36 μm/h; clone 14, 27.79 ± 4.77 μm/h), the heterozygous cells (clone 4, 26.98 ± 3.33 μm/h) and wild-type C2C12 cells (26.49 ± 2.70 μm/h). These results demonstrate that LRRC8A, and hence VRAC activity, plays no important role in C2C12 migration in the wound healing assay.

### 2.2. VRAC Blockers and Disruption of LRRC8s Do Not Impair HCT 116 Proliferation and Migration

**Figure 2:**
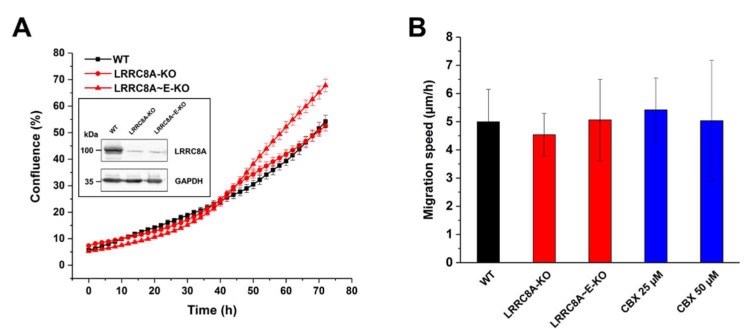
Effect of LRRC8 subunit knockout or carbenoxolone (CBX) treatment on cell proliferation and migration of HCT116 cells. (**A**) Growth curve of wild-type (WT), LRRC8A-knockout (KO), and LRRC8A~E-knockout (KO) HCT116 cells. Data represent mean ± SD of *n* = 6–9 experiments. Inset: Knockout of the LRRC8A subunit was confirmed by Western blotting. (**B**) Effect of LRRC8 subunits knockout or treatment with CBX on migration of HCT116 cells. Cell migration speed was determined 24 h post-wounding. Data represent mean ± SD of *n* = 7 experiments.

Since the involvement of ion channels in cell growth and migration is of particular interest in relation to cancer progression [45,46,47,48], we investigated a potential role of VRAC in the proliferation and migration of human colon cancer HCT116 cells. We first examined the effects of genomic VRAC knockout on HCT116 proliferation (Figure 2A). Although the proliferation of genomic VRAC knockout clones seemed slightly decreased as compared with wild-type cells during the first 48 h, the proliferation of a clonal cell line lacking the essential LRRC8A subunit of VRAC was virtually equal to that of wild-type cells over the complete time course. Another clonal cell line, lacking all five LRRC8 members, even displayed an increase in proliferation. These results demonstrate that VRAC is not critically involved in HCT116 proliferation. Next, we examined the effect of the genomic VRAC deletion and of the VRAC inhibitor carbenoxolone (CBX) on HCT116 cell motility in our wound healing assay (Figure 2B). Neither pharmacological inhibition of VRAC with up to 50 µM CBX, nor gene knockout of VRAC affected motility of the HCT116 cells. Together, these data demonstrate that VRAC is dispensable for human colon cancer proliferation and migration.

### 2.3. LRRC8A/VRAC Is Not Required for the Proliferation and Migration of Glioblastoma Cells

**Figure 3:**
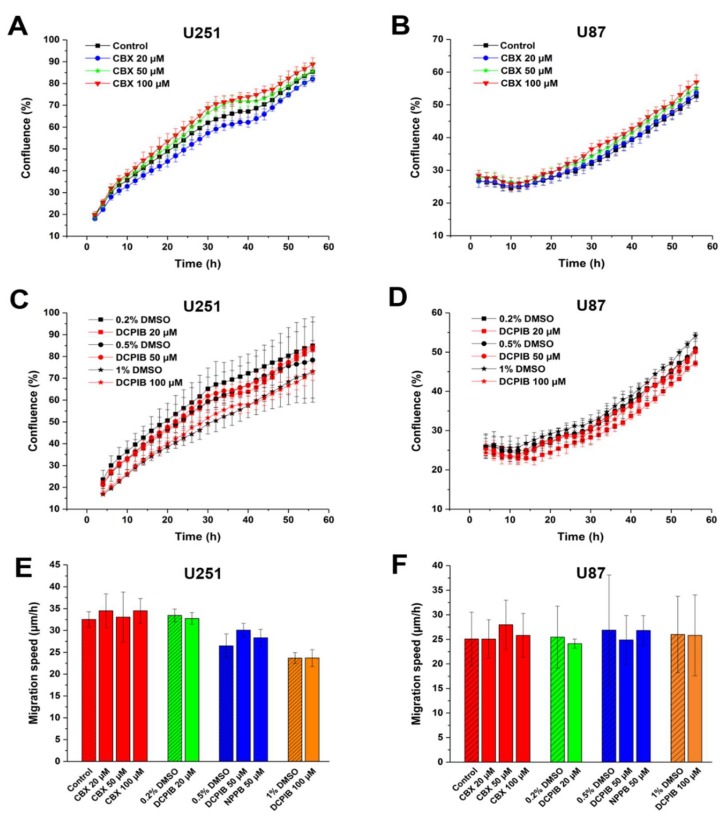
Volume-regulated anion channel (VRAC) blockers do not affect proliferation and migration of glioblastoma multiforme (GBM) cells. Growth curve of U251 (**A**,**C**) and U87 (**B**,**D)** after treatment with indicated concentrations of CBX (**A**,**B**) or DCPIB (**C**,**D**). Data represent mean ± SD of *n* = 3–7 experiments. Cell migration of U251 (**E**) and U87 (**F**) after treatment with indicated concentrations of CBX, NPPB or DCPIB. The diagonal lines represent controls. Data represent mean ± SD of *n* = 4–10 experiments.

While VRAC plays no important role in HCT116 cell proliferation and migration, the contribution of VRAC to cell proliferation and migration may vary between cell types. Glioblastoma multiforme (GBM) is a common, rapidly growing malignant brain tumor [49,50]. To examine the contribution of VRAC to GBM cell proliferation and migration, we first assessed the effects of pharmacological inhibitors on the established glioblastoma cell lines U251 and U87 (Figure 3). Treatment with up to 100 µM CBX did not alter the proliferation rate of U251 or 87 cells (Figure 3A,B). Consistently, proliferation was neither affected by VRAC inhibition with up to 100 µM DCPIB (Figure 3C,D). Next, we tested the effect of the VRAC inhibitors on GBM cell migration in the wound healing assay. We observed no significant differences in migration speed between inhibitor-treated and control U251 and U87 cells (Figure 3E,F). Collectively, these results suggest that VRAC activity is dispensable for GBM cell proliferation and 2D migration.

**Figure 4:**
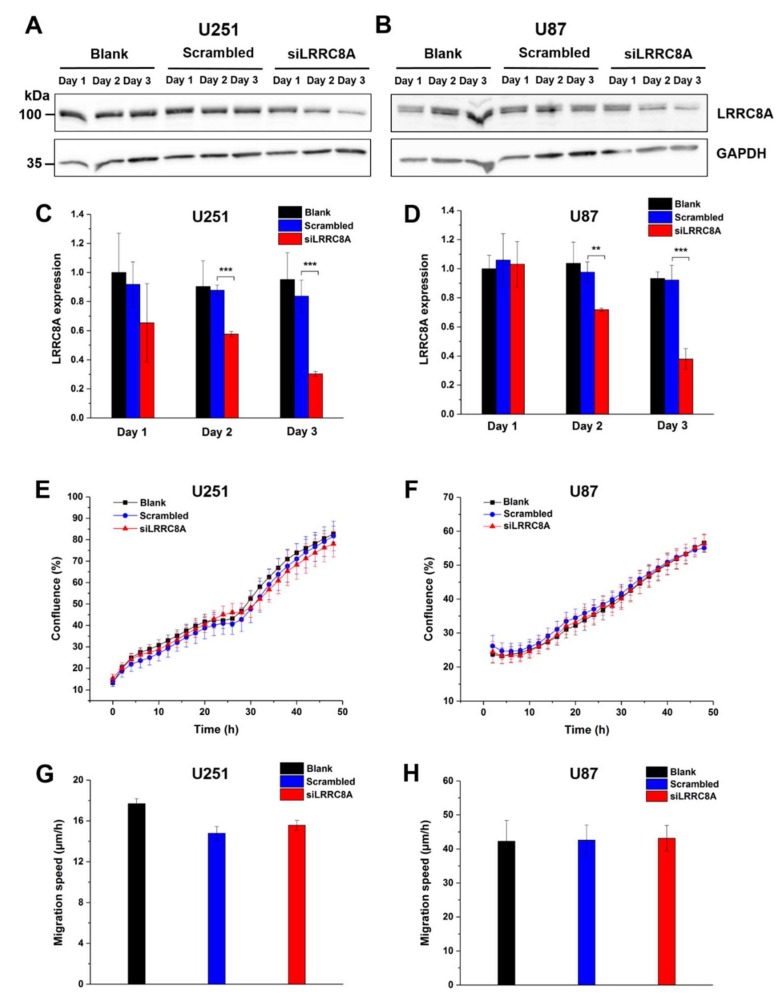
Downregulation of LRRC8A does not affect GBM cell growth and motility. Expression of LRRC8A protein significantly decreased after U251 (**C**) and U87 (**D**) cells transfected with siRNA against LRRC8A for 48 h and 72 h. ** *p* < 0.01, *** *p* < 0.001 vs. scrambled. Data represent mean ± SD from *n* = 3 independent experiments. GAPDH on the same blot was used as an internal control. Representative blots of three independent experiments for U251 (**A**) and U87 (**B**) were shown. Proliferation of U251 (**E**) and U87 (**F**) started 48 h after transfection with siRNA against LRRC8A. Data represent mean ± SD of *n* = 8 experiments. (**G**) U251 cell migration started after 48 h transfection with siRNA against LRRC8A. The data were calculated after cell migrated for 24 h. Data represent mean ± SD of *n* = 5 experiments. (**H**) U87 cell migration started after 48 h transfection with siRNA against LRRC8A. Note the high values due to absence of mitomycin in the wound healing assay in this case. The data were calculated after cell migrated for 14 h. Data represent mean ± SD of *n* = 7 experiments.

Since these data are in apparent conflict with the previously reported effect of DCPIB on GBM cell migration [28], we additionally approached the role of VRAC by silencing the expression of the essential VRAC subunit LRRC8A with siRNA. Western blotting confirmed a robust reduction of LRRC8A protein amount after transfection with siRNA against LRRC8A for 48 or 72 h in U251 and U87 cells at two days (by roughly 40% and 30%, respectively) and three days (by roughly 70%) after transfection with siRNA against LRRC8A (Figure 4A–D) cells. Proliferation of both cell lines, assessed from 48 h after siRNA or control transfection onwards, was not affected by the LRRC8A knockdown. In the wound healing assay, also started 48 h after transfection, we observed no significant differences in the migration speed between non-transfected cell, cells transfected with control siRNA, and cells transfected with siRNA against LRRC8A at various time points after transfection (Figure 4G,H, Figure S1).

Together, these results from pharmacological VRAC inhibition and LRRC8A knockdown suggest that VRAC is dispensable for glioblastoma cell proliferation and migration in the wound healing assay.

### 2.4. VRAC Inhibition by DCPIB or LRRC8A Downregulation Does Not Affect PI3K/Akt Signaling in GBM Cells

**Figure 5:**
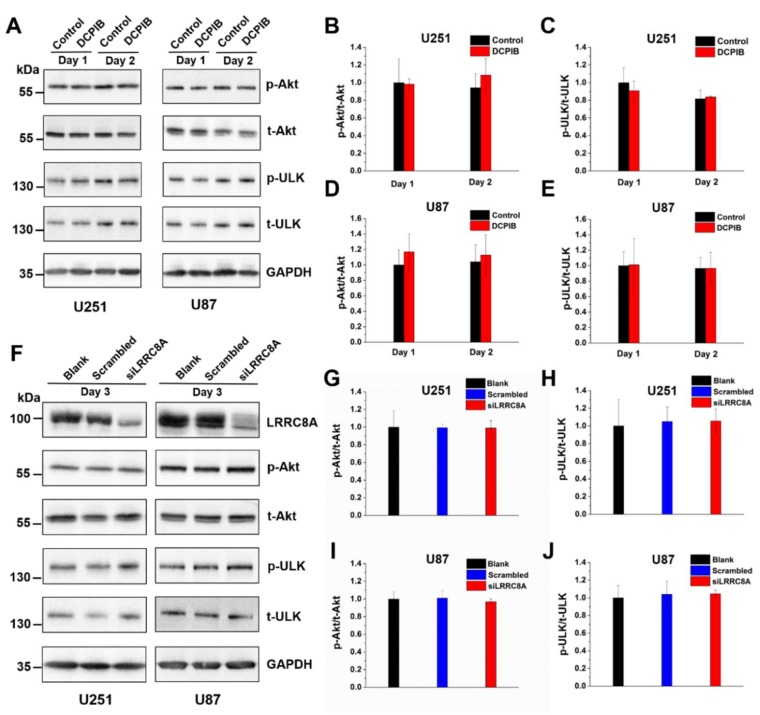
Treatment with DCPIB or siRNA-mediated knockdown of LRRC8A have no effects on PI3K/Akt signaling. (**A**) Western blot of GBM cells treated with DCPIB (100 μM) at day 1 or day 2 for total amount and phosphorylated forms of Akt and ULK. GAPDH used as loading control. A representative blot of three independent experiments is shown. Analysis of the *p*-Akt/t-Akt (**B**,**D**) and *p*-ULK/t-ULK (**C**,**E**) ratio for U251 (**B**,**C**) and U87 (**D**,**E**) cells. (**F**) Western blotting of GBM cells for total amount and phosphorylated forms of Akt and ULK 72 h after siRNA transfection. For LRRC8A, the same samples were used as in Figure 4A and 4B. GAPDH used as loading control. A representative blot of three independent experiments is shown. Analysis of the *p*-Akt/t-Akt (**G**,**I**) and *p*-ULK/t-ULK (**H**,**J**) ratio for U251 (**G**,**H**) and U87 (**I**,**J**) cells. All data are presented as mean ± SD of *n* = 3 independent experiments.

Since activation of mTOR signaling by the PI3K/Akt pathway is involved in the regulation of GBM cell proliferation and migration [51,52,53], we examined whether VRAC is involved in PI3K/Akt/mTOR signaling. To this end, we assessed the phosphorylation status of Akt and the mTOR substrate ULK by Western blotting (Figure 5). Treatment of U251 or U87 GBM cells with 100 µM DCPIB for one or two days did not alter the relative phosphorylation of Akt or ULK (*p*-Akt/t-Akt and *p*-ULK/t-ULK, respectively, Figure 5A–E). Likewise, the phosphorylation was not changed three days after LRRC8A siRNA transfection, when LRRC8A protein levels were significantly reduced (Figure 5H,J). Collectively, the results suggest that neither pharmacological VRAC inhibition nor siRNA-mediated downregulation of LRRC8A affected PI3K/Akt signaling.

## 3. Discussion

The volume-regulated anion channel (VRAC) is ubiquitously expressed in vertebrate cells [1,2,29] and contributes to regulatory volume decrease upon osmotic cell swelling. As the extracellular osmolarity is usually kept constant, most animal cells rarely experience extracellular hypo-osmolarity under normal conditions. Thus, the VRAC is thought to play roles in other physiological processes by its impact on cellular volume, such as during cell proliferation and migration [2,26,54].

Several studies reported an impairment of proliferation and/or migration of various cell lines in the presence of VRAC inhibitors [24,25,28,29,30,31,32,33,34,35,36,37,38]. However, the available VRAC inhibitors display little selectivity and often also inhibit other anion channels [55,56], or as in the case of the potent and relatively selective VRAC blocker DCPIB [57] even modulate potassium channels [58,59]. The identification of LRRC8 proteins as essential VRAC components [4,5] enabled investigating physiological functions of VRAC by molecular biological tools. Using this approach, siRNA-mediated downregulation of the essential VRAC subunit LRRC8A reduced proliferation of primary glioblastoma and U251 GBM cells [40], and knockdown of LRRC8A in the colorectal cancer cell line HCT116 was shown to impair cell migration in a wound healing assay [41].

In contrast, using both pharmacological and molecular biological approaches we found no evidence for a role of VRAC in the proliferation or migration in a range of cell lines including non-differentiated C2C12 myoblasts, colorectal cancer HCT116 cells, and GBM cell lines U251 and U87. Whereas, inhibition of VRAC with DCPIB (at higher concentrations than required to inhibit VRAC currents) in these latter GBM cell lines [28] and siRNA-mediated LRRC8A knockdown in U251 cells [40] was reported to reduce cell viability and proliferation, however, we did not observe such effect by either treatment in these cell lines. A possible explanation for the apparently conflicting results may be that we measured the increasing confluence of the proliferating cells, while in the previous studies the cells’ metabolic activity was measured using the MTT assay. Measuring the cell number directly with a Coulter counter, the most efficient siRNA showed much less reduction in cell proliferation as compared with the MTT assay [40]. The reportedly reduced migration of GBM cells in the presence of DCPIB, which we did not observe in our study, may be explained by their impaired proliferation also during the wound healing assay [28]. The discrepancy between our finding, on the one hand, that knockout of LRRC8A or all LRRC8 members did not diminish HCT116 cell migration, and on the other hand, the previous report of slowed HCT116 migration upon siRNA-mediated LRRC8A knockdown [41] is unlikely due to upregulation of compensatory mechanisms in our case, since the migration speed was also unaffected upon acute pharmacological VRAC inactivation.

Other studies corroborate the notion that VRAC is dispensable for cell proliferation. The flavonoid Dh-morin suppressed VRAC currents but did not reduce proliferation of HUVEC cells [43], and siRNA against LRRC8A did not affect the proliferation rate in HeLa cells [42]. The DCPIB treatment even inhibited the antiproliferative effect of cardiac glycosides that correlated with an increase in VRAC activity in HT-29 cells [44].

The slowed proliferation and migration of U251 GBM cells was related to a reduction of PI3K/Akt/mTor signaling in the presence of 100 µM DCPIB for 24 or 48 h [28]. In our study, we were not able to detect differences in the basal phosphorylation state of Akt1 in U251 or U87 GBM cells upon treatment with 100 µM DCPIB for one or two days. Neither did we find a reduced phosphorylation of the mTOR substrate ULK. In addition, Akt signaling was not altered by siRNA-mediated LRRC8A knockdown. This was consistent with the previously reported normal anti-CD3-mediated activation of Akt1 in thymocytes from LRRC8A-deficient mice [60]. In adipocytes, LRRC8A was reported to be involved in the regulation of insulin-stimulated Akt2 signaling [61]. However, no effect on Akt1 was detected upon LRRC8A deletion in that study [61], which was in agreement with our findings for glioblastoma cells.

In summary, our study demonstrates that LRRC8/VRAC is not crucially involved in general cell proliferation. Its indispensability for cell migration in the wound healing assay does not rule out a role for VRAC in constricted environments where directed osmotic water flux has been shown to drive cellular locomotion [23]. The VRAC inhibitor NPPB was shown to impair this process of cell invasion [62]. Apart from VRAC, other chloride channels may contribute, such as the calcium-activated chloride channel TMEM16A [63]. Future work is required to clarify the potentially cell-type-specific roles of the different types of ion channels under more physiological conditions in cell migration and invasion.

## 4. Materials and Methods

### 4.1. Reagents

Mitomycin C was purchased from Sigma-Aldrich (Taufkirchen, Germany). 5-nitro-2-(3-phenylpropylamino)benzoic acid (NPPB) and 4-[(2-Butyl-6,7-dichloro-2-cyclopentyl-2,3-dihydro-1-oxo-1H-inden-5-yl)oxy]butanoic acid (DCPIB) were purchased from Tocris Bioscience (Avonmouth, Bristol, UK). Carbenoxolone (CBX) and fibronectin were purchased from Sigma-Aldrich. Dimethyl sulfoxide (DMSO) was purchased from PAN-Biotech (Aidenbach, Germany).

### 4.2. Cell Culture

Wild-type and LRRC8 knockout HCT116 cell lines (clone 3F8 for *LRRC8A*^-/-^) [4], kindly provided by T. J. Jentsch (FMP and MDC, Berlin, Germany), were maintained in McCoy’s 5A Medium (GIBCO) with 10% fetal bovine serum (FBS) at 37 °C in the presence of antibiotics in a humidified atmosphere with 5% CO_2_. U251 and U87 cells were kindly provided by U. Stein and H. Kettenmann (both MDC, Berlin, Germany), respectively, and authenticated using highly-polymorphic short tandem repeat loci (Microsynt, Balgach, Switzerland). C2C12 cells, kindly provided by P. Knaus (Freie Universität Berlin, country), U251 and U87 cells were maintained in Dulbecco’s Modified Eagle Medium (DMEM) (Sigma-Aldrich) supplemented with 10% FBS, 100 U/mL penicillin, and 100 μg/mL streptomycin in a 37 °C, 5% CO_2_ humidified chamber.

### 4.3. siRNA Transfection

U251 and U87 cells were plated in 6-well cell culture plates (1.5 × 10^5^ cells per well) 1 day prior to transfection. The cell culture medium was removed and cells were washed with the serum-free Opti-MEM, then transfected with siRNA against LRRC8A (*Lrrc8a* siRNA: sense-CCU UGU AAG UGG GUC ACC ATT) (ThermoFisher Scientific, Darmstadt, Germany) #s109501 at a concentration of 15 nM using lipofectamine RNAiMax transfecting agent (ThermoFisher Scientific). A nontargeting, scrambled siRNA (ThermoFisher Scientific, 4390844) was used as a negative control. For the proliferation assay, cells were grown for a further 48 h before seeding into a 96-well plate. For the migration assay, cells were grown for a further 30–42 h post-transfection, then plated in a 96-well ImageLock™ tissue culture plate. Wounds were created 48 h after siRNA transfection.

### 4.4. Cell Proliferation and Migration Assays

To assess cell proliferation, 5000 cells (10,000 cells in the case of HCT116) per well were seeded into a 96-well plate and placed into the IncuCyte live-cell analysis system. Before scanning, the plate was allowed to acclimatize for 30 min. Cell proliferation was monitored by using the IncuCyte system (Sartorius, Göttingen, Germany) to capture phase contrast images every 2 h during constant incubation at 37 °C in a humidified atmosphere with 5% CO_2_.

To assess cell migration, 2–8 × 10^4^ cells (depending on cell type) per well were seeded into a 96-well ImageLock™ plate (Essen BioScience 4379, Sartorius, Göttingen, Germany) and incubated for 4–16 h at 37 °C in a humidified atmosphere with 5% CO_2_ before replacing the medium with culture medium supplemented with 5 µg/mL mitomycin C. Mitomycin C was applied during the following steps, if not specified otherwise, to inhibit cell proliferation so that this process would not distort our results on cell migration. After 2 h, wounds were created in all wells of the 96-well ImageLock™ plate with the WoundMaker™ (Sartorius, Göttingen, Germany). After gently washing the wells twice with culture medium, 100 μL of medium containing additional drugs (CBX, DCPIB, NPPB, or vehicle DMSO when appropriate) were applied to each well. NPPB and DCPIB were dissolved in DMSO, CBX in water. Cell migration was monitored by phase contrast imaging with an IncuCyte Zoom microscope, acquiring an image every 2 h during constant incubation at 37 °C in a humidified atmosphere with 5% CO_2_. The IncuCyte Zoom image analysis software (Sartorius, Göttingen, Germany) was used to detect cell edges automatically and to generate an overlay mask for wound width calculation. For the migration assay with HCT116, the 96-well ImageLock™ plates were coated with fibronectin prior to cell seeding.

### 4.5. Antibodies and Western Blotting

For Western blotting, equivalent amounts of sample protein were separated by 10% SDS-PAGE and transferred to nitrocellulose membrane (Macherey Nagel, Düren, Germany). After blocking with 5% BSA in TBS-T (20 mM Tris pH 7.6, 150 mM NaCl and 0.02% Tween-20), the membrane was immunoblotted with primary antibodies overnight at 4 °C. The following primary antibodies were used: rabbit anti-LRRC8A (1 μg/mL, [4] kindly supplied by T. J. Jentsch); rabbit anti-p-ULK (1:1000, Cell Signaling, Frankfurt am Main, Germany; #14202); rabbit anti-ULK (1:1000, cell signaling, #8054); rabbit anti-p-Akt^Ser473^ (1:1000, Cell Signaling; #4060); rabbit anti-Akt^pan^ (1:1000, Cell Signaling; #4685); and rabbit anti-GAPDH (1:2500, Cell Signaling; #2118) antibodies. The corresponding peroxidase-labeled secondary antibody (1:10,000, Jackson ImmunoResearch, Ely, UK) was used. Signals were detected using an enhanced chemiluminescence reagent (HRP juice; PJK GmbH, Kleinblittersdorf, Germany) and a ChemiSmart5000 digital imaging system (Vilber-Lourmat, Collégien, France). Densitometrical quantification was performed with the Fiji software [64].

### 4.6. Generation of C2C12 LRRC8A Knockout Cell Lines Using CRISPR/Cas9 Technology

To create LRRC8A knockout C2C12 cells using the CRISPR/Cas9 technology, the targeting sgRNA sequences (5′-GCCCCGGAAGGAGTCGTTGC*AGG*-3′) was cloned into the px459-V.2 vector and transfected into C2C12 cells. Two days post-transfection, transfected cells were selected by treatment with 10 µg/mL puromycin for two days before single clone expansion by dilution to statistically 0.5 cells per well in 96-well format. Monoclonal cell lines were expanded and tested for sequence alterations using target-site-specific PCR with primers 5′-CATGTATGTCTCACTACACCTAACTTGTAG-3′ and 5′-CCAGGAAGATGAGGGTGTGCA-3′ on genomic DNA followed by Sanger sequencing.

### 4.7. Statistical Analysis

Proliferation and migration were quantified with the IncuCyte Zoom image analysis software by measuring cell confluence and wound width over time, respectively. The software OriginPro 2017 (OriginLab, Northampton, MA, USA) was used for statistical analyses. All data are presented as the mean values ± SD For comparisons between two groups, *p*-values were determined using Student’s *t*-test and are indicated according to convention: * *p* < 0.05, ** *p* < 0.01 and *** *p* < 0.001.

## Supplementary Materials

Supplementary Materials can be found at https://www.mdpi.com/1422-0067/20/11/2663/s1.

## Abbreviations

CBX	Carbenoxolone
DCPIB	4-[(2-Butyl-6,7-dichloro-2-cyclopentyl-2,3-dihydro-1-oxo-1H-inden-5-yl)oxy]butanoic acid
GBM	Glioblastoma multiforme
HUVEC	Human umbilical vein endothelial
NPPB	5-nitro-2-(3-phenylpropylamino)benzoic acid
RVD	Regulatory volume decrease
RVI	Regulatory volume increase
VRAC	Volume-regulated anion channel
VSOR	Volume-sensitive outwardly rectifying
