# Supplementary material for: The Volume-Regulated Anion Channel LRRC8/VRAC Is Dispensable for Cell Proliferation and Migration
Source: Int J Mol Sci. 2019 May 30;20(11):2663. doi: 10.3390/ijms20112663 (PMC6600467; doi:10.3390/ijms20112663)
Supplement: Supplementary file 1 [file ijms-20-02663-s001.pdf]

Supplementary information for

The volume-regulated anion channel LRRC8/VRAC is dispensable for cell proliferation and migration

Tianbao Liu and Tobias Stauber

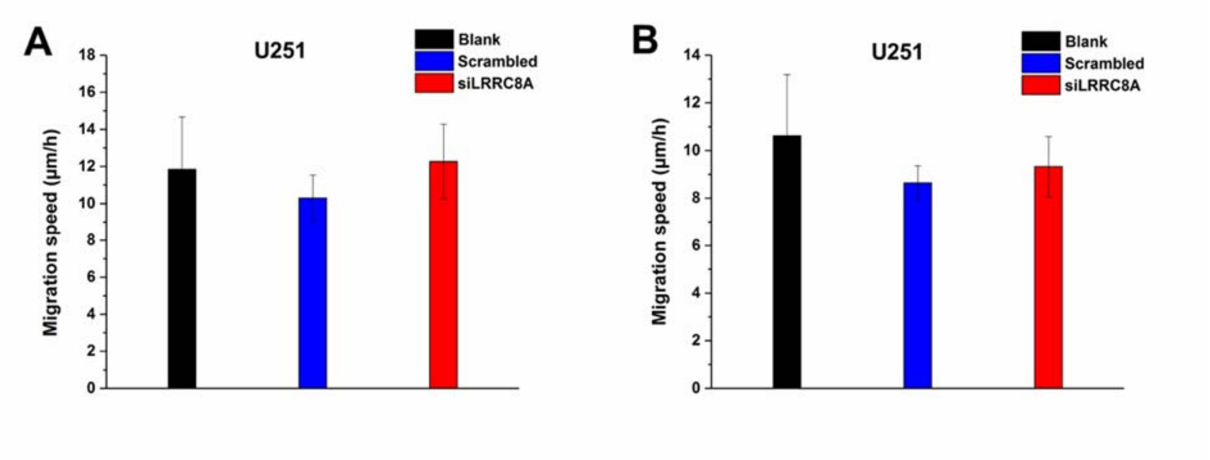

Figure S1. siRNA-mediated knockdown of LRRC8A has no significant effect on U251 cell migration, independent of the migration time. U251 cell migration started 48 h after transfection with siRNA against LRRC8A. The data were calculated after cells migrated for 36 h (**A**) or 48 h (**B**). All data represent mean ± SD of n = 5 experiments.
